# A natural agonist of mosquito TRPA1 from the medicinal plant *Cinnamosma fragrans* that is toxic, antifeedant, and repellent to the yellow fever mosquito *Aedes aegypti*

**DOI:** 10.1371/journal.pntd.0006265

**Published:** 2018-02-09

**Authors:** Edna Alfaro Inocente, Marguerite Shaya, Nuris Acosta, L. Harinantenaina Rakotondraibe, Peter M. Piermarini

**Affiliations:** 1 Department of Entomology, The Ohio State University, Ohio Agricultural Research and Development Center, Wooster, Ohio, United States of America; 2 Departments of Medicinal Chemistry and Pharmacognosy, The Ohio State University, Columbus, Ohio, United States of America; Johns Hopkins University, Bloomberg School of Public Health, UNITED STATES

## Abstract

Plants produce various secondary metabolites that offer a potential source of novel insecticides and repellents for the control of mosquito vectors. Plants of the genus *Cinnamosma* are endemic to, and widely-distributed throughout, the island of Madagascar. The barks of these species are commonly used in traditional medicines for treating a wide range of maladies. The therapeutic nature of the bark is thought to be associated with its enrichment of pungent drimane sesquiterpenes, which elicit antifeedant and toxic effects in some insects. Here we test the hypothesis that a bark extract of *Cinnamosma fragrans* (CINEX) and its major drimane sesquiterpenes are insecticidal, antifeedant, and repellent to *Aedes aegypti*, the principal mosquito vector of chikungunya, dengue, yellow fever, and Zika viruses. We demonstrate that CINEX is 1) toxic to larval and adult female mosquitoes, and 2) antifeedant and repellent to adult female mosquitoes. Moreover, we show that cinnamodial (CDIAL), a sesquiterpene dialdehyde isolated from CINEX, duplicates these bioactivities and exhibits similar toxic potency against pyrethroid-susceptible and -resistant strains of *Ae*. *aegypti*. Importantly, we show that CDIAL is an agonist of heterologously-expressed mosquito Transient Receptor Potential A1 (TRPA1) channels, and the antifeedant activity of CDIAL is dampened in a TRPA1-deficient strain of *Ae*. *aegypti* (*TRPA1-/-*). Intriguingly, *TRPA1-/-* mosquitoes do not exhibit toxic resistance to CDIAL. The data indicate that modulation of TRPA1 is required for the sensory detection and avoidance of CDIAL by mosquitoes, but not for inducing the molecule’s toxicity. Our study suggests that CDIAL may serve as a novel chemical platform for the development of natural product-based insecticides and repellents for controlling mosquito vectors.

## Author summary

New insecticides and repellents are urgently needed to improve the control of mosquito vectors of emerging diseases, such as Zika. Here we discover that the bark of a Madagascan medicinal plant (*Cinnamosma fragrans*) is enriched with a molecule (cinnamodial) that is toxic, antifeedant, and repellent to the principal mosquito vector of Zika virus (*Aedes aegypti*). We also show that the mechanism behind some of cinnamodial’s activity in mosquitoes is associated with its ability to activate a receptor (TRPA1) that mosquitoes use to detect heat and noxious chemicals in their environment. The results from our work may lead to the development of new insecticides and chemicals that can be used to control mosquito vectors.

## Introduction

New insecticides and repellents are needed to diversify our chemical arsenal for controlling mosquito vectors of emerging pathogens, such as Zika virus. Plants produce a remarkable array of secondary metabolites that are a valuable source of chemical scaffolds with potential insecticidal and/or repellent activities [1]. Plant species of the genus *Cinnamosma* (family Canellaceae; *Cinnamosma fragrans*, *C*. *macrocarpa*, *C*. *madagascariensis*) are endemic to—and widely-distributed throughout—the island of Madagascar. Decoctions of the barks of these species are commonly used in traditional medicines as a ‘cure-all’ for treating a wide range of conditions, including malaria, general fatigue, and muscle aches [2–4]. The therapeutic nature of the bark is thought to be related to its high content of drimane sesquiterpenes, which are known to possess anti-plasmodial, anti-hyperalgesic, and anti-nociception properties [5–8]. Drimane sesquiterpenes are secondary metabolites found not only in several families of higher plants (e.g., Canellaceae, Winteraceae, Polygonaceae, and Asteraceae), but also in some liverworts, fungi, molluscs, and sponges, where they play an important role in chemical defense against potential predators and pathogenic microorganisms [9–11]. Notably, several plant-derived drimane sesquiterpenes are toxic and/or antifeedant to insects [12–16], suggesting they have potential uses as insecticides and/or repellents.

**Fig 1:**
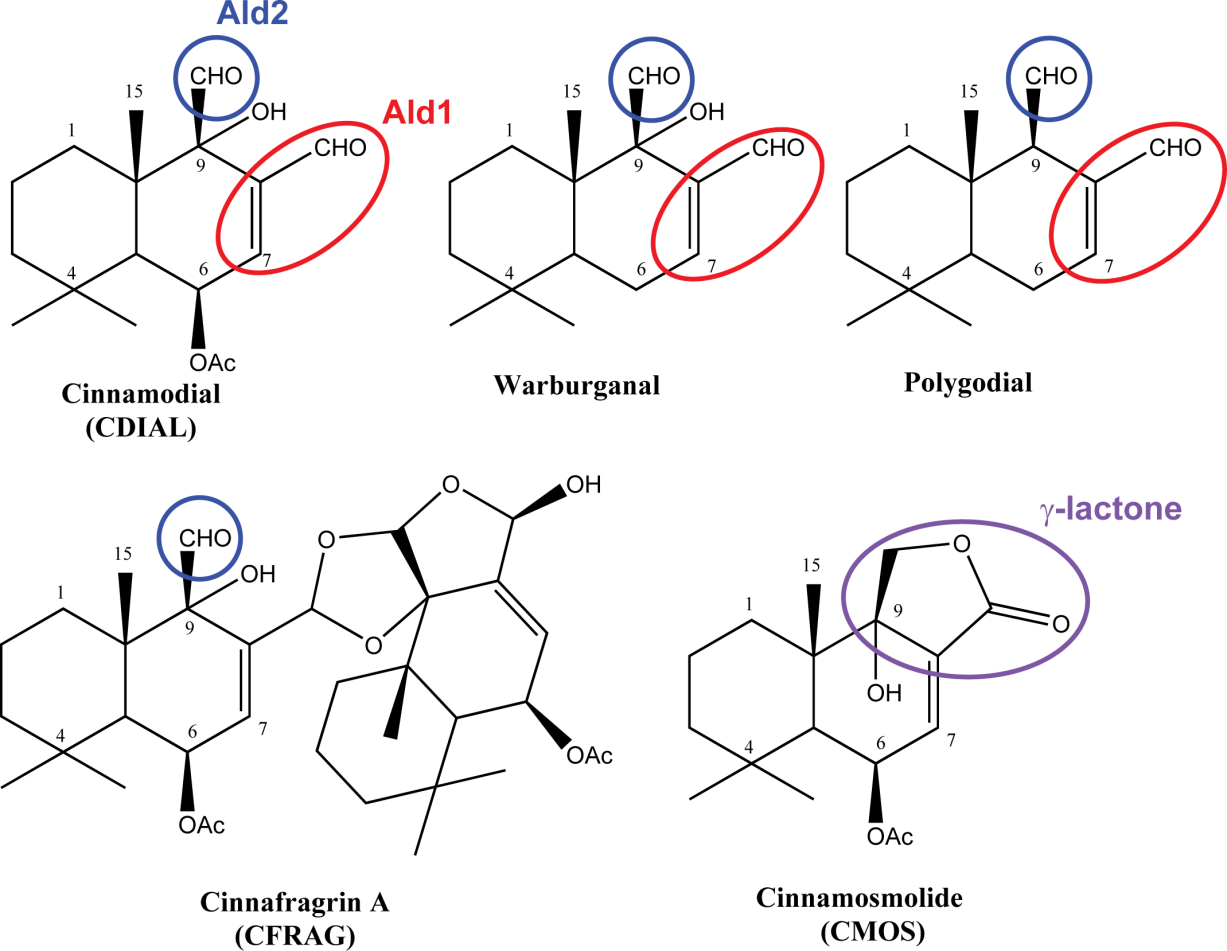
Structural comparison of representative drimane sesquiterpenes. CDIAL, CFRAG, and CMOS were the focus of the present investigation. The conjugation of Ald1 with the double-bond at C-7 and C-8 makes CDIAL a strong electrophile (S1 Fig). The presence of Ald2 attached to a quaternary carbon (C-9) bearing a hydroxyl group also contributes to the electrophilic nature of the molecule (S1 Fig).

For the past several years, we have focused on isolating, determining the structures of, and characterizing the bioactivities of drimane sesquiterpenoids from the bark and leaves of *C*. *fragrans*, which is a widely used plant in Madagascan traditional medicine. In the dichloromethane extract of the bark of *C*. *fragrans* (CINEX), cinnamodial (CDIAL; Fig 1) is one of the most abundant compounds, representing about 65% of the sesquiterpenoids [17]. CDIAL is characterized by the presence of two electrophilic aldehyde functions; Ald1 is a strong electrophile while Ald2 is a relatively weak electrophile (Fig 1, S1 Fig). The structure of CDIAL is very similar to other drimane sesquiterpenes with reported toxic and antifeedant activity against insects, such as warburganal and polygodial (Fig 1). Apart from CDIAL, other less electrophilic sesquiterpenes have been isolated from *C*. *fragrans* and its congeners, including: cinnafragrin A (CFRAG), a dimer of CDIAL with only the non-conjugated aldehyde function (i.e., Ald2); and cinnamosmolide (CMOS), a derivative of CDIAL where the aldehyde functions are replaced with a γ-lactone ring (Fig 1) [17–19]. Both CDIAL and CMOS are active in a remarkable variety of bioassays. In particular, they: 1) inhibit α-glucosidase activity [17, 18]; 2) are cytotoxic to various cancer cell lines [18, 20–22]; 3) are antifungal [23]; and 4) exhibit vanilloid-like activity [24, 25]. In addition, CDIAL inhibits: 1) growth of murine leukemia cells, human T-lymphocytes, and bacteria [17, 26]; 2) mammalian inflammatory mechanisms [27]; and 3) feeding activity of lepidopteran pests [16, 28, 29].

Intrigued by the antifeedant activity of CDIAL against lepidopterans [16, 28, 29], the toxicity of similar drimane sesquiterpenes against insects [12–16], and a recent finding that essential oils from the leaves and bark of *C*. *madagascariensis* were toxic to larval *Culex quinquefasciatus* [30], we tested the hypothesis that CINEX and its major drimane sesquiterpenes (CDIAL, CFRAG, and CMOS) were insecticidal, antifeedant, and repellent to mosquitoes. Focusing on the yellow fever mosquito *Aedes aegypti*—the principal vector of Zika, dengue, chikungunya, and yellow fever viruses—we demonstrated that CINEX was toxic to larval and adult female mosquitoes, and antifeedant and repellent to adult female mosquitoes. Importantly, we identified CDIAL as the primary molecule responsible for the bioactivities of CINEX against mosquitoes.

Emerging evidence in vertebrate and insect systems (including mosquitoes) suggests that electrophilic unsaturated sesquiterpene dialdehydes are agonists of Transient Receptor Potential A1 (TRPA1) channels, which are receptors that play important roles in the environmental sensing and avoidance of potentially noxious chemicals, as well as temperatures [31–39]. Chemical agonists of TRPA1 have been suggested as potential molecules for the development of new insect repellents [31, 34, 40–43]. Thus, we also tested the hypothesis that CDIAL’s mechanism of action in mosquitoes involved the modulation of TRPA1. We found that: 1) CDIAL was an agonist of heterologously-expressed mosquito TRPA1; and 2) the antifeedant activity, but not the toxicity, of CDIAL was dampened in a TRPA1-deficient strain of *Ae*. *aegypti*. Taken together, the results of the present study indicate that CDIAL may serve as a valuable chemical platform for the development of next-generation, natural product-based insecticides and repellents for controlling mosquito vectors.

## Methods

### Plant materials, and extraction, isolation and structure determination of sesquiterpenoids

The root bark of *Cinnamosma fragrans* was collected on public land in the Commune Lakato/Alaotra Mangoro region of Madagascar. The extraction of the plant and isolation of the compounds (CDIAL, CMOS, and CFRAG) were performed according to our previous work [19]. The structures of CDIAL, CFRAG, and CMOS were confirmed by comparing their ^1^H and ^13^C NMR spectroscopic data with those reported in the literature [19].

### Mosquito cultures and rearing conditions

Eggs of the Liverpool (LVP) and Puerto Rico (PR) strains of *Aedes aegypti* were obtained through the MR4 as part of the BEI Resources Repository, NIAID, NIH (LVP-IB12, MRA-735, deposited by M.Q. Benedict; PR, NR-48830, deposited by G.G. Clark & J.J. Becnel). Eggs of the Orlando (ORL) and *TRPA1-/-* (*Aaeg*TRPA1^ECFP-2^) [31] strains of *Ae*. *aegypti* were generously provided by the laboratory of Dr. Leslie Vosshall (Rockefeller University). The genetic status of the *TRPA1-/-* mutants used in the present study was confirmed using an established diagnostic PCR-based approach [31]. Eggs of *Culex pipiens* were obtained from a recently established laboratory colony at The Ohio State University [44], and eggs of *Anopheles gambiae* (G3 strain) were obtained through BEI Resources, NIAID, NIH (G3, MRA-112; contributed by M.Q. Benedict). The eggs of the *Ae*. *aegypti* and *Cx*. *pipiens* strains were reared to adults as described previously [44, 45]. The eggs of *An*. *gambiae* were reared to adults similarly to *Ae*. *aegypti*, but baker’s yeast (*Saccharomyces cerevisiae*) was added to the larval diet. To maintain the pyrethroid-resistance trait of the PR strain, 3^rd^ instar larvae were treated en masse with 0.1 mg/ml cypermethrin (Acros Organics, Geel, Belgium) for 10–15 min (until ~50% of larvae stopped swimming) every third generation.

### Toxicology assays

The larval toxicities of CINEX, the isolated sesquiterpenes, and cypermethrin were evaluated using an established assay [46, 47]. In brief, five 1^st^-instar larvae were added to the wells of a 24-well Falcon Multiwell plate (Becton Dickinson Labware, Franklin Lakes, NJ) containing 985 μl of dH_2_O and 5 μl of a food solution (13 mg/ml of finely ground fish food flakes in dH_2_O; Tetramin, Blacksburg, VA). To each well, 10 μl of CINEX, a sesquiterpene, or cypermethrin (all dissolved in 100% acetone) at various concentrations was added. Control wells received 10 μl of 100% acetone. The plates were held under normal rearing conditions (28°C, 80% relative humidity, 12:12 light:dark) for 24 h before assessment of larvae. The efficacy of a concentration was determined as the percentage of larvae in a well that died within 24 h. Larvae were counted as dead if they did not move after gentle touching with a fine needle or pipette tip.

The adult toxicities of CINEX and the isolated sesquiterpenes were evaluated using an established assay [46, 48]. In brief, groups of 10 adult female mosquitoes (3–10 days post-emergence) were immobilized on ice and treated with 500 nl (*Ae*. *aegypti*, *Cx*. *pipiens*) or 200 nl (*An*. *gambiae*) of a compound (dissolved in 100% acetone) at various concentrations. The compounds were delivered to the thorax of mosquitoes with a repeating dispenser (PB600-1, Hamilton, Reno, NV). As a control, 100% acetone was used. Immediately after treatment, the mosquitoes were transferred to small cages (32 oz. containers) with access to 10% sucrose and held under normal rearing conditions for 24 h before assessment. The efficacy of a dose was defined as the percentage of incapacitated mosquitoes (i.e., dead or unable to fly) in a cage within 24 h [46, 48–51].

The concentration/dose-response curves for CINEX, the sesquiterpenes, and cypermethrin were evaluated with GraphPad Prism (version 6.07) software. In brief, percent efficacies were plotted against the log transformations of the concentrations/doses. The EC_50_, ED_50_, and Hill slope values were determined with non-linear regressions using the log(agonist) vs. normalized response function. Statistical comparisons of the best fit values were made with F-tests using GraphPad Prism software.

### Antifeedant assays

The antifeedant activities of CINEX and the isolated sesquiterpenes were assessed with a capillary feeding (CAFE) choice assay, originally designed for adult *Drosophila melanogaster* [52] and later adapted to adult female *Ae*. *aegypti* by the Vosshall laboratory [31]. Prior to the assay, adult female mosquitoes (3–10 days post-emergence) were starved of 10% sucrose for 24 h, but given access to water. For an experiment, groups of five mosquitoes were placed in *Drosophila* vials (28.5 x 95 mm; VWR International, Radnor, PA) and covered with cotton plugs. Two holes were added to each plug to allow for the insertion of 5-μl calibrated glass capillaries (VWR International). The bottoms of the capillaries were carefully placed so that they did not protrude more than 1 mm into the vial from the plug to ensure mosquitoes could access the liquid while landed on the plug. One capillary was designated the control and filled with 5 μl of 10% sucrose containing 0.01% trypan blue (to provide contrast) and 1% acetone (the solvent of the compounds). The other capillary was designated the treatment and filled with 5 μl of 10% sucrose containing 0.01% trypan blue and CINEX (0.48 mg/ml) or an isolated sesquiterpene (0.75 or 1.5 mM). These concentrations were chosen because they were shown to elicit consistent antifeedant effects with CINEX and/or CDIAL in preliminary trials. The concentrations of sesquiterpenes used were below and similar, respectively, to those used in previous studies assessing the antifeedant activities of *N*-methylmaleimide (10 mM) and kinin analogs (0.1–1.0 mM) in *Ae*. *aegypti* [31, 53]. The tops of the capillaries were capped with mineral oil to minimize evaporative losses. In some vials, the capillaries were filled with identical control sucrose solutions containing 1% acetone to confirm there was no inherent feeding bias in the assay. Vials without mosquitoes, but containing the filled capillaries, were also included to account for evaporative losses.

All experimental vials were placed in normal rearing conditions for 18–20 h (starting between 3:00 and 4:00 PM) after which the volume of sucrose remaining in each capillary was measured. The difference between the starting and ending volumes was the consumption volume (after correcting for evaporative losses). Antifeedant activity was calculated using the approach of Isman et al. [54]; i.e., the volume consumed from the treatment capillary was subtracted from that of the control capillary and divided by the total volume consumed from both capillaries. The resulting activity value was multiplied by 100 and expressed as a ‘percent’. The mean antifeedant activity values were compared using GraphPad Prism (version 6.07) software with a one-way ANOVA and Holm-Sidak’s multiple comparisons test.

### Repellency assays

To assess the repellencies of CINEX and the isolated sesquiterpenes we developed a membrane blood-feeding, no-choice assay. Twenty-four hours prior to an experiment, groups of 20 adult female mosquitoes (3–7 days post-emergence) were placed in small plastic cages (32 oz. containers) covered with a taught mesh screen and given access to water. All experiments were performed between 1:00 and 4:00 PM. For an experiment, a feeding disc of a Hemotek membrane feeder (Blackburn, UK) was placed on top of the mesh screen for one hour under normal rearing conditions. The feeding disc (5.77 cm^2^ surface area) was filled with defibrinated rabbit blood (HemoStat Laboratories, Dixon, CA) containing 0.01 g/ml of adenosine 5’-triphosphate (ATP) as a feeding stimulant, and warmed to 37°C to attract mosquitoes. The bottom of each disc, which faced the mesh screen, consisted of a collagen membrane (treated with 10% lactic acid as another attractant) that acted as a reservoir for the blood. After filling the feeding discs with blood, nylon fabric (No nonsense Regular Pantyhose, Kayser-Roth Corporation, Greensboro, NC) was stretched over the collagen membrane and 250 μl of CINEX (0.48 mg/ml), DEET (0.48 mg/ml = 2.5 mM), or an isolated sesquiterpene (1.5 mM), each dissolved in 100% acetone, was applied to the fabric/membrane. Controls received 250 μl of 100% acetone. These concentrations were chosen because they were shown to elicit consistent repellent effects with CINEX and CDIAL in preliminary trials. The nylon fabric facilitated the spread of acetone evenly across the feeding membrane and provided an additional substrate for the compounds to adhere. The treated discs (with nylon and membrane) were allowed to dry at room temperature (~5 min) before placing them on the mesh screen.

After a 1 h feeding period, the mosquitoes were immobilized by submerging the cages in ice for 5–10 min. The abdomen of each mosquito in the cage was visually inspected for the ingestion of blood to calculate the percentage of mosquitoes that fed. Mosquitoes with any visibly detectable blood in their abdomens were considered fed. The percent reduction in the number of mosquitoes that fed from the treatment cage (relative to the mean feeding percentage of the control cages) was calculated and expressed as a ‘percent mosquitoes repelled’. The mean values among treatments were compared using GraphPad Prism (version 6.07) software with a one-way ANOVA and Holm-Sidak’s multiple comparisons test. The mean percentage of mosquitoes that blood fed in the control cages was 75.2 ± 2.2% (N = 35 cages of 20 mosquitoes each).

### Heterologous expression and two-electrode voltage clamping in *Xenopus laevis* oocytes

Defolliculated *Xenopus laevis* oocytes (Ecocyte Bioscience, Austin, TX) were injected with 28 nl of *An*. *gambie* (*Ag*) TRPA1 cRNA (1 ng/nl) and cultured in OR3 media for 3–5 days at 18°C. Control oocytes were injected with 28 nl of nuclease-free H_2_O. The *Ag*TRPA1 cRNA was generated with a mMessage mMachine SP6 Transcription kit (Ambion, Thermo Fisher Scientific, Waltham, MA) using an *Ag*TRPA1 cDNA as a template. The *Ag*TRPA1 cDNA was previously cloned [34] and generously provided by the laboratory of Dr. Laurence J. Zwiebel (Vanderbilt University). The encoded amino-acid sequence of *Ag*TRPA1 is ~84% identical to that of the closest ortholog in *Ae*. *aegypti* (AAEL009419).

Two-electrode voltage clamping experiments were performed similarly to those in Piermarini et al [55]. Whole-cell currents were acquired and recorded with an OC-725 oocyte clamp (Warner Instruments) bridged to a Windows PC running pCLAMP software (Axoscope, Version 10, Molecular Devices) via a MiniDigi-1A interface (Molecular Devices). For a given experiment, an oocyte was placed in a RC-3Z chamber (Warner Instruments) under superfusion (2 ml/min) with ND96 solution [56] and impaled with two electrodes filled with 3 M KCl. Once the resting membrane potential (V_m_) stabilized (~1–2 min), the V_m_ was clamped to a hyperpolarizing potential of 30 mV relative to the resting V_m_ to promote inward (negative) membrane currents (I_m_).

Once the oocyte was voltage clamped, it was superfused with an experimental ND96 solution containing CDIAL, CFRAG, or CMOS (10 μM). After 2 min, the superfusion was switched back to normal ND96 solution to wash out the experimental compound for 2 min. The superfusion was then switched to ND96 solution containing ruthenium red (10 μM) for 2 min to end the experiment. All solutions were delivered by gravity to the oocyte chamber using polyethylene tubing, and the solution changes were performed with a Rheodyne Teflon 8-way Rotary valve (Model 5012, Rheodyne, Rohnert Park, CA). The maximal changes in I_m_ associated with the addition of a compound (ΔI_m_) and the rate at which the changes in I_m_ occurred (ΔI_m_/Δt) were calculated using pCLAMP software (Clampfit, Version 10, Molecular Devices). The mean ΔI_m_ and ΔI_m_/Δt values were statistically compared using GraphPad Prism (version 6.07) software with a one-way ANOVA and Holm-Sidak’s multiple comparisons test, and unpaired t-test respectively.

## Results

### Toxicity to larval and adult female mosquitoes

**Fig 2:**
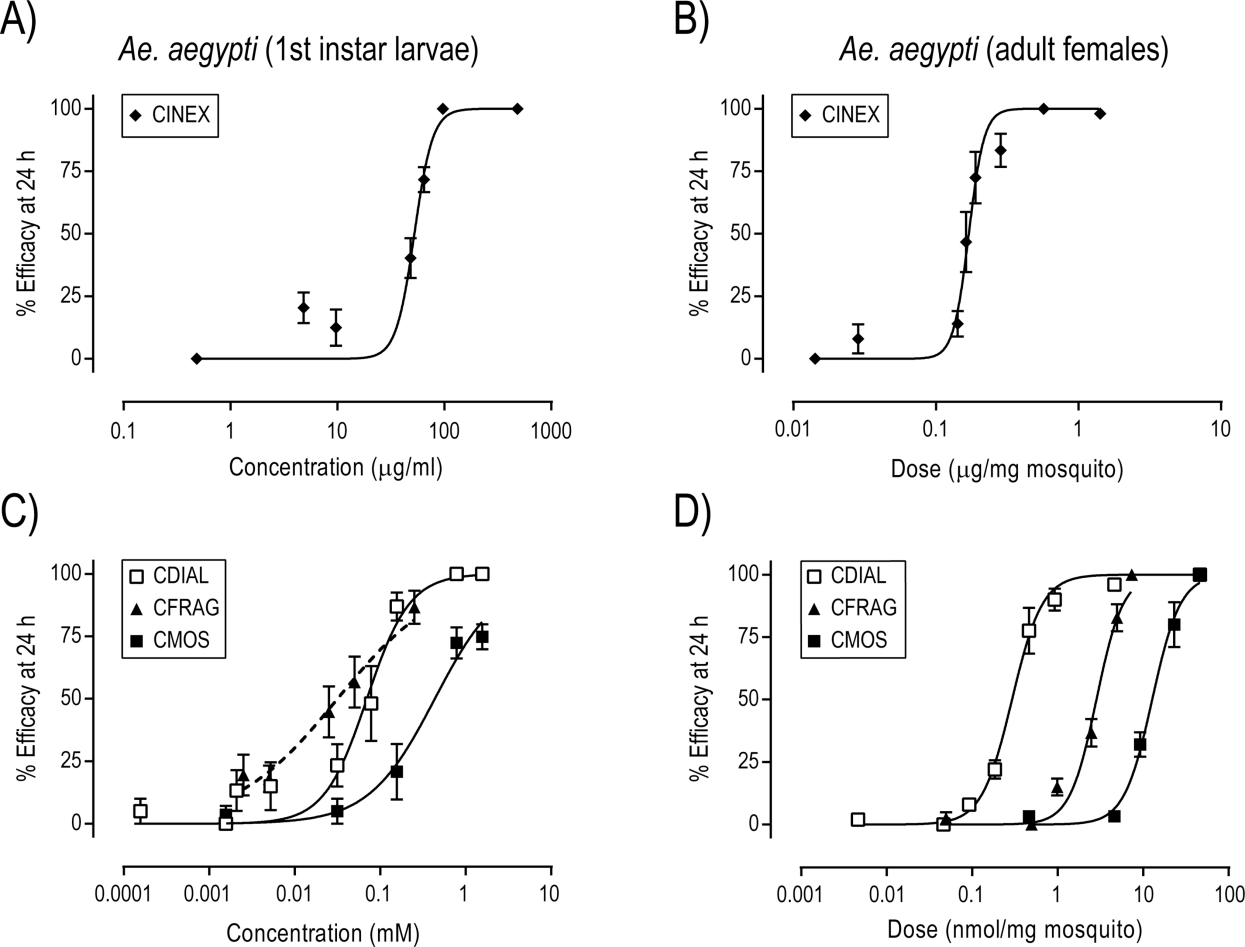
Toxicity of CINEX and isolated sesquiterpenes to *Ae*. *aegypti* (LVP strain). A,C) Concentration-toxicity relationships in 1^st^ instar larvae 24 h after adding CINEX or indicated compound to the rearing water. Efficacy was defined as the percentage of larvae that were dead within 24 h. Values are means ± SEM based on 3–8 independent replicates of 5 larvae per concentration. The mean efficacy of the acetone controls was 0.43 ± 0.43% (N = 46 independent replicates of 5 larvae). B,D) Dose-toxicity relationships in adult females 24 h after applying CINEX or indicated compound to the thoracic cuticle. Efficacy was defined as the percentage of adults that were incapacitated (dead or flightless) within 24 h. Values are means ± SEM based on 3–8 independent replicates of 10 females per dose. The mean efficacy of the acetone controls was 0.67 ± 0.37% (N = 45 independent replicates of 10 females). See text or Table 1 for the specific potency values (EC_50_ or ED_50_) and Hill slopes.

We first assessed the toxicity of CINEX against larval and adult female *Ae*. *aegypti* (Liverpool, LVP, strain). As shown in Fig 2A, adding CINEX to the rearing water of 1^st^ instar larvae elicited concentration-dependent toxicity within 24 h (EC_50_ = 52.5 μg/ml, 95% CI = 48.0–57.5 μg/ml; Hill slope = 5.06, 95% CI = 1.77–8.35). Likewise, topical application of CINEX to the thoracic cuticle of adult females resulted in dose-dependent toxicity within 24 h (Fig 2B; ED_50_ = 0.17 μg/mg, 95% CI = 0.16–0.18 μg/mg; Hill slope = 8.03, 95% CI = 4.41–11.64).

Previous studies and NMR profiling of CINEX have shown that CDIAL, CFRAG and CMOS were major constituents, with CDIAL as the most abundant [17–19]. Thus, we assessed their respective toxicities against mosquitoes. As shown in Fig 2C, CDIAL was the only compound to elicit concentration-dependent toxicity in larvae that reached 100% efficacy within 24 h (EC_50_ = 70 μM, 95% CI = 53.8–91.0 μM; Hill slope = 1.74, 95% CI = 0.97–2.56). CFRAG reached a maximal efficacy of ~85% at ~250 μM while CMOS only reached a maximal efficacy of ~75% at ~1.5 mM (Fig 2C). Higher concentrations of CFRAG and CMOS could not be tested due to their limited solubility in acetone and/or the larval rearing water. In adult females, CDIAL, CFRAG, and CMOS each exhibited dose-dependent topical toxicity that reached 100% efficacy (Fig 2D). The ED_50_ value of CDIAL was ~10- and ~44-times lower than that of CFRAG and CMOS, respectively, and the Hill slopes of the compounds were all similar (Fig 2D, Table 1). Thus, CDIAL was the most toxic to adult females, followed by CFRAG and CMOS.

**Table 1:** Dose-toxicity parameters of sesquiterpenes isolated from CINEX on adult females of *Ae*. *aegypti* (LVP strain). * = significantly different from CDIAL as determined by a F-test (P < 0.05).

	ED_50_ in nmol/mg mosquito (95% CI)	Hill slope (95% CI)
CDIAL	0.29 (0.25–0.34)	2.45 (1.78–3.11)
CFRAG	2.85* (2.52–3.22)	2.79 (1.98–3.595)
CMOS	12.79* (11.20–14.61)	2.64 (1.95–3.33)

* = significantly different from CDIAL as determined by a F-test (P < 0.05).

**Fig 3:**
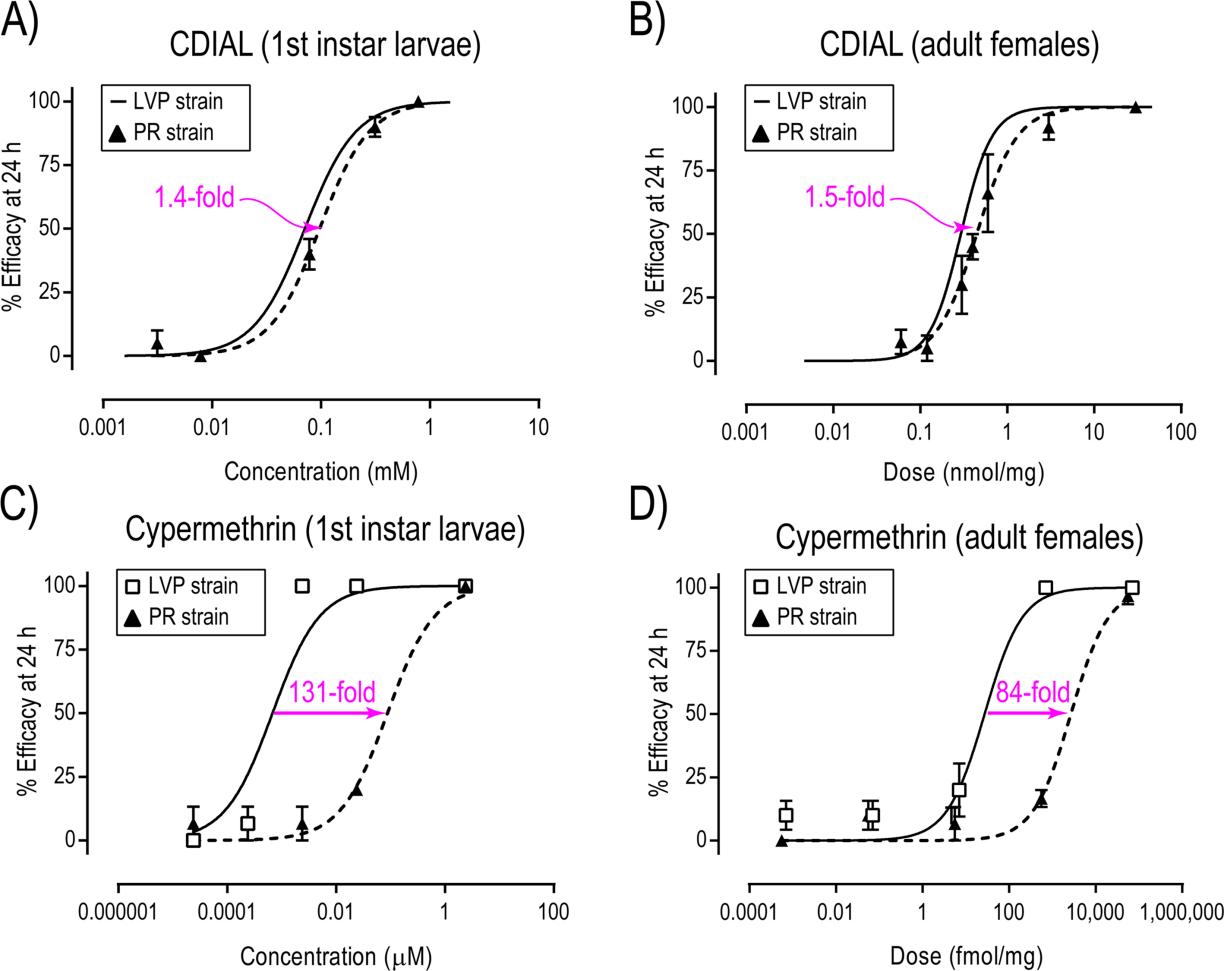
**Comparative toxic resistance of a pyrethroid-resistant strain of *Ae*. *aegypti* (Puerto Rican strain, PR) to CDIAL (A, B) vs. cypermethrin (C,D).** The concentration/dose-toxicity relationships of CDIAL against the LVP strain of *Ae*. *aegypti* are from Fig 2 (data points are omitted for clarity). A,C) Concentration-toxicity relationships in 1st instar larvae 24 h after adding indicated compound to the rearing water. Efficacy was defined as the percentage of larvae that were dead within 24 h. Values are means ± SEM based on 3–12 independent replicates of 5 larvae per concentration. The EC_50_ of CDIAL in PR (97.1 μM; 95% CI = 82.4–114.5 μM) was slightly (1.4-fold), but significantly (P < 0.05; F-test), greater than that in LVP (70 nM; 95% CI = 53.8–91.0 μM). The EC_50_ of cypermethrin in PR (88.0 nM; 95% CI = 46.2–167.7 nM) was dramatically (131-fold) and significantly (P < 0.05; F-test) greater than that in LVP (0.68 nM; 95% CI = 0.34–1.34 nM). B,D) Dose-toxicity relationships in adult females 24 h after applying indicated compound to the thoracic cuticle. Efficacy was defined as the percentage of adults that were incapacitated (dead or flightless) within 24 h. Values are means ± SEM based on 3–8 independent replicates of 10 females per dose. The ED_50_ of CDIAL in PR (0.45 nmol/mg; 95% CI = 0.34–0.58 nmol/mg) was slightly (1.5-fold), but significantly, (P < 0.05; F-test) greater than that in LVP (0.29 nmol/mg; 95% CI = 0.25–0.34 nmol/mg). The ED_50_ of cypermethrin in PR (2,685.0 fmol/mg; 95% CI = 1315.0–5484.0 fmol/mg) was dramatically (84-fold) and significantly (P < 0.05; F-test) greater than that in LVP (27.3 fmol/mg; 95% CI = 12.1–61.4 fmol/mg).

Parallel experiments with CDIAL in larvae and adult females of pyrethroid-resistant *Ae*. *aegypti* (Puerto Rico, PR, strain) revealed a weak (≤ 1.5-fold), but significant (P < 0.05; F-test), resistance relative to the LVP strain (Fig 3A and 3B). In contrast, larvae and adult females of the PR strain were strongly (≥ 84-fold) and significantly (P < 0.05; F-test) resistant to the pyrethroid cypermethrin relative to the LVP strain (Fig 3C and 3D). Parallel experiments in larvae and adult females of *Cx*. *pipiens* and *An*. *gambiae* confirmed the toxicity of CDIAL to other medically-important mosquito vectors (S2 Fig).

### Antifeedant activity in adult female mosquitoes

**Fig 4:**
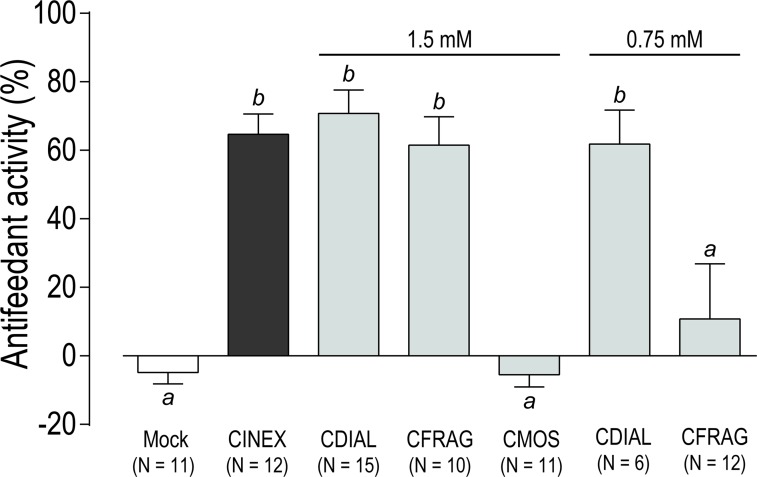
Antifeedant activity of CINEX and isolated sesquiterpenes as determined via choice CAFE assays in adult female *Ae*. *aegypti* (LVP strain). Groups of 5 mosquitoes were allowed to feed equally on two capillaries of 10% sucrose with 0.01% trypan blue; the control capillary included 1% acetone (the solvent), and the treatment capillary included 1% acetone and CINEX (0.48 μg/μl) or a sesquiterpene (0.75 or 1.5 mM). In ‘Mock’ experiments, both capillaries included 1% acetone alone. The difference in volume consumed between the capillaries was used to calculate the antifeedant activity (see Methods for details). Values are means ± SEM; N = number of independent replicates of 5 mosquitoes each. Lower-case letters indicate statistical categorization of the means as determined by a one-way ANOVA and Holm-Sidak’s posttest (P < 0.05).

To assess whether CINEX, CDIAL, CFRAG, and CMOS were antifeedant to adult female *Ae*. *aegypti* (LVP strain), we used a capillary feeding (CAFE) choice bioassay [31, 52]. In brief, 5 mosquitoes were presented with two capillaries of 10% sucrose as a food source for 18–20 h; the ‘control’ capillary was treated with 1% acetone (the solvent for CINEX and the compounds) and the ‘treatment’ capillary was treated with CINEX or an isolated sesquiterpene. To provide a baseline for comparison and ensure there was no inherent bias to the assay, a ‘mock’ experiment was performed where only the solvent was added to both capillaries; in this case, the mosquitoes fed from each capillary equally, resulting in nominal antifeedant activity (‘Mock’ in Fig 4). However, if the treatment capillary contained 0.48 μg/μl CINEX, 1.5 mM CDIAL, or 1.5 mM CFRAG, then the mosquitoes consumed more sucrose from the control capillary, resulting in significant antifeedant activity compared to the mock (Fig 4). No significant antifeedant activity was elicited when the treatment capillary contained 1.5 mM CMOS (Fig 4). The significant antifeedant activity of CDIAL, but not CFRAG, persisted when the concentration was reduced to 0.75 mM (Fig 4), suggesting that CDIAL was a more potent antifeedant than CFRAG.

### Repellency against adult female mosquitoes

**Fig 5:**
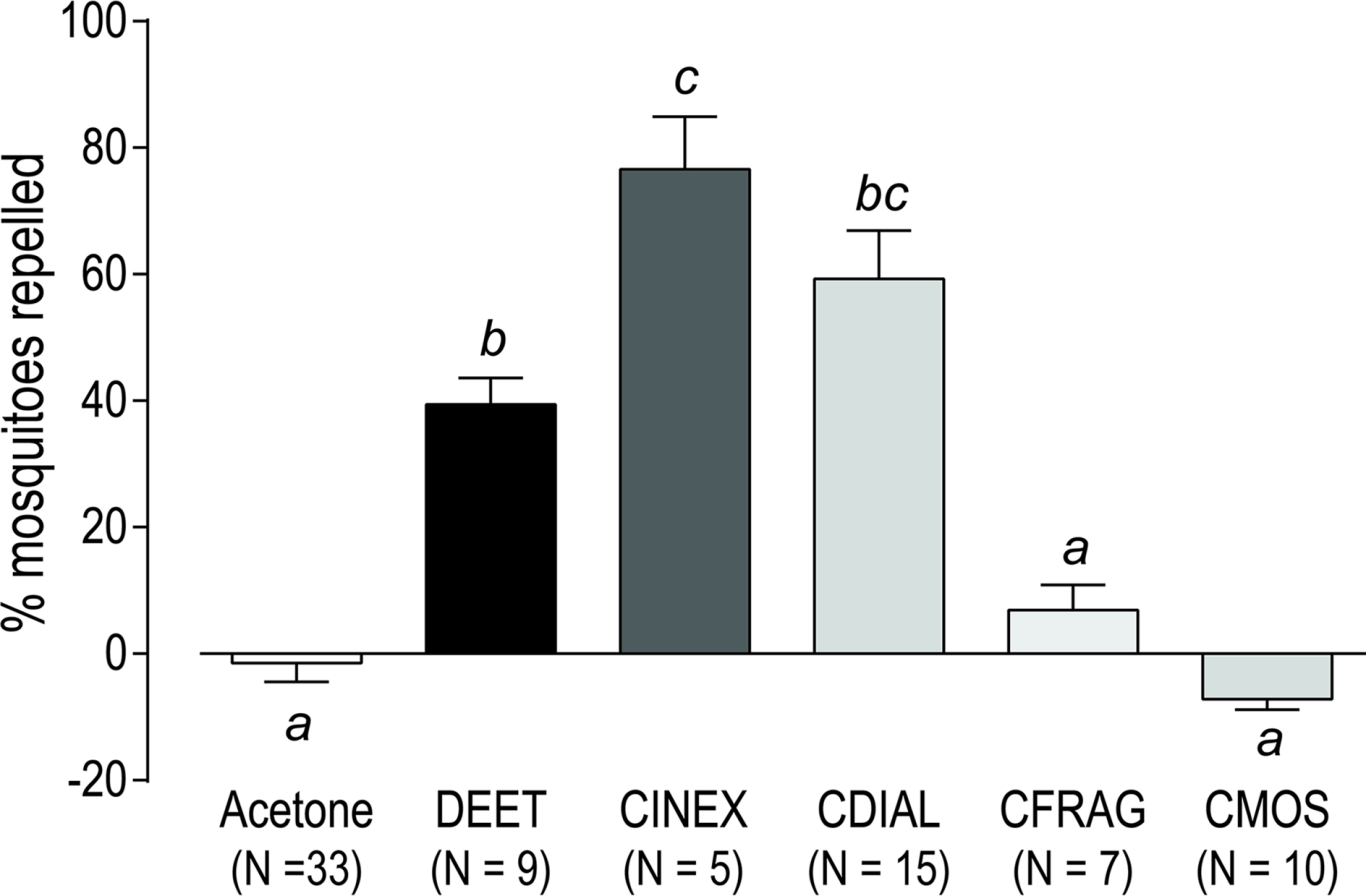
Repellency of DEET, CINEX, and isolated sesquiterpenes in adult female *Ae*. *aegypti* (LVP strain) as determined via a non-choice membrane blood-feeding bioassay. Cages of 20 mosquitoes were allowed to feed on a blood source (defibrinated rabbit blood) for 1 h; the feeding membrane was treated with DEET (20.8 μg/cm^2^ = 108.75 nmol/cm^2^), CINEX (20.8 μg/cm^2^), or a sesquiterpene (67.45 nmol/cm^2^) dissolved in 100% acetone. Control membranes were treated with 100% acetone. The number of mosquitoes that fed from each cage was determined. The percent reduction in the number of mosquitoes that fed from the treatment cage (relative to the mean feeding percentage of the control cages) was calculated to determine ‘percent mosquitoes repelled’. Values are means ± SEM; N = number of independent replicates of 20 mosquitoes each. Lower-case letters indicate statistical categorization of the means as determined by a one-way ANOVA and Holm-Sidak’s posttest (P < 0.05).

To determine whether CINEX, CDIAL, CFRAG, and CMOS repelled blood-seeking adult female *Ae*. *aegypti* (LVP strain), we developed a membrane blood-feeding, no-choice bioassay (see Methods for details). As shown in Fig 5, treating the membrane feeder’s surface with 20.8 μg/cm^2^ DEET (108.75 nmol/cm^2^), a positive control, significantly repelled ~40% of the mosquitoes from feeding on blood compared to the 100% acetone (solvent) control. Notably, treating the surface with 20.8 μg/cm^2^ CINEX was ~2-times more effective than DEET (Fig 5). Furthermore, CDIAL (67.45 nmol/cm^2^) significantly repelled ~60% of the mosquitoes from feeding on blood (Fig 5). On the other hand, CFRAG and CMOS (67.45 nmol/cm^2^) did not significantly repel mosquitoes compared to the acetone control (Fig 5).

### Modulation of mosquito TRPA1 by CDIAL, CFRAG, and CMOS

We next tested whether CDIAL, CFRAG, and CMOS modulated the activity of heterologously-expressed *Ag*TRPA1 in *Xenopus* oocytes [34]. We used *Ag*TRPA1 as a representative mosquito TRPA1, because the ortholog from *Ae*. *aegypti* (AAEL009419) had not been previously cloned and the encoded amino-acid sequence of *Ag*TRPA1 is ~84% identical to that predicted for AAEL009419.

**Fig 6:**
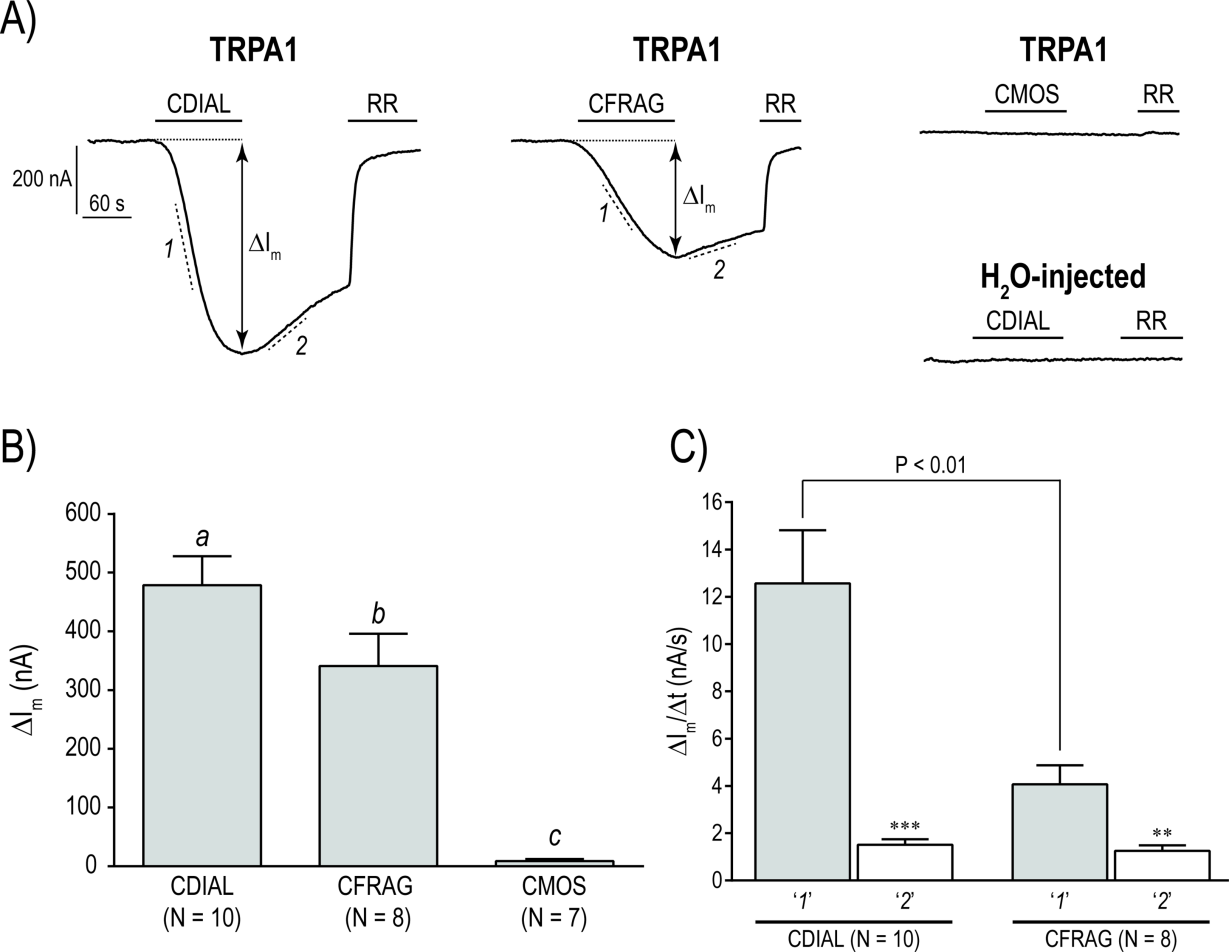
Effects of isolated sesquiterpenes on the electrophysiological activity of *Xenopus* oocytes expressing *Ag*TRPA1 as determined by two-electrode voltage clamping. A) Representative traces of membrane current (I_m_) in *Ag*TRPA1-expressing (TRPA1) or H_2_O-injected oocytes. Horizontal bars indicate the addition of 10 μM CDIAL, CFRAG, CMOS, or ruthenium red (RR) to the extracellular bath. The bidirectional arrows show the peak changes in membrane current (ΔI_m_). The dashed lines indicate the activation (*1*) and deactivation (*2*) rates (ΔI_m_/Δt). For H_2_O-injected oocytes, the addition of CDIAL (shown), CFRAG (S3 Fig), or CMOS (S3 Fig) did not noticeably elicit a change in I_m_. B) Summary of ΔI_m_ elicited by each sesquiterpene (10 μM) in TRPA1-expressing oocytes. Values are means ± SEM; N = number of oocytes measured. Lower-case letters indicate statistical categorization of the means as determined by a one-way ANOVA and Holm-Sidak’s posttest (P < 0.05). C) Summary of ΔI_m_/Δt during periods ‘*1*’ and ‘*2*’ shown in panel A for CDIAL and CFRAG. Values are means ± SEM; N = number of oocytes measured. The mean ΔI_m_/Δt during period ‘*1*’ for CDIAL was significantly greater than that for CFRAG as determined by an unpaired t-test (P < 0.01). ‘***’ and ‘**’ indicate significant difference (P < 0.001 and P < 0.01, respectively) between activation (‘*1*’) and deactivation (‘*2*’) rates for each molecule as determined by a paired t-test.

Fig 6A shows representative tracings of membrane current (I_m_) in voltage-clamped oocytes injected with *Ag*TRPA1 cRNA or H_2_O. Adding 10 μM CDIAL to the extracellular bath elicited a prominent inward (negative) I_m_, reflecting the opening of *Ag*TRPA1 cation channels (Fig 6A). CFRAG produced a similar response, but the peak I_m_ elicited by CDIAL (ΔI_m_ in Fig 6A) was larger in magnitude and manifested more rapidly (slope ‘*1*’ in Fig 6A). In contrast, 10 μM CMOS elicited a nominal ΔI_m_ (Fig 6A). On average, CDIAL manifested the largest ΔI_m_ in *Ag*TRPA1 oocytes, followed by CFRAG and CMOS (Fig 6B), and the activation rate of *Ag*TRPA1 by CDIAL (slope ‘*1*’; ΔI_m_/Δt) was significantly faster (P < 0.01) than that by CFRAG (Fig 6C). Thus, CDIAL was the most effective agonist of *Ag*TRPA1, followed by CFRAG and CMOS.

The subsequent washing out of CDIAL or CFRAG reversed the trajectory of I_m_ towards the baseline (slope ‘*2*’ in Fig 6A), but at a slower rate than the corresponding activation (compare ‘*1*’ vs. ‘*2*’ in Fig 6A). On average, the activation rates of *Ag*TRPA1 by CDIAL and CFRAG were significantly faster than their associated deactivation rates (‘*1*’ vs ‘*2*’ in Fig 6C). On the other hand, addition of ruthenium red (10 μM) to the bath, a generic blocker of TRP channels, promptly returned I_m_ to baseline values (‘RR’ in Fig 6A). In H_2_O-injected oocytes, adding CDIAL, CFRAG, or CMOS to the bath did not noticeably affect I_m_ (e.g., ‘H_2_O-injected’ in Fig 6A).

**Fig 7:**
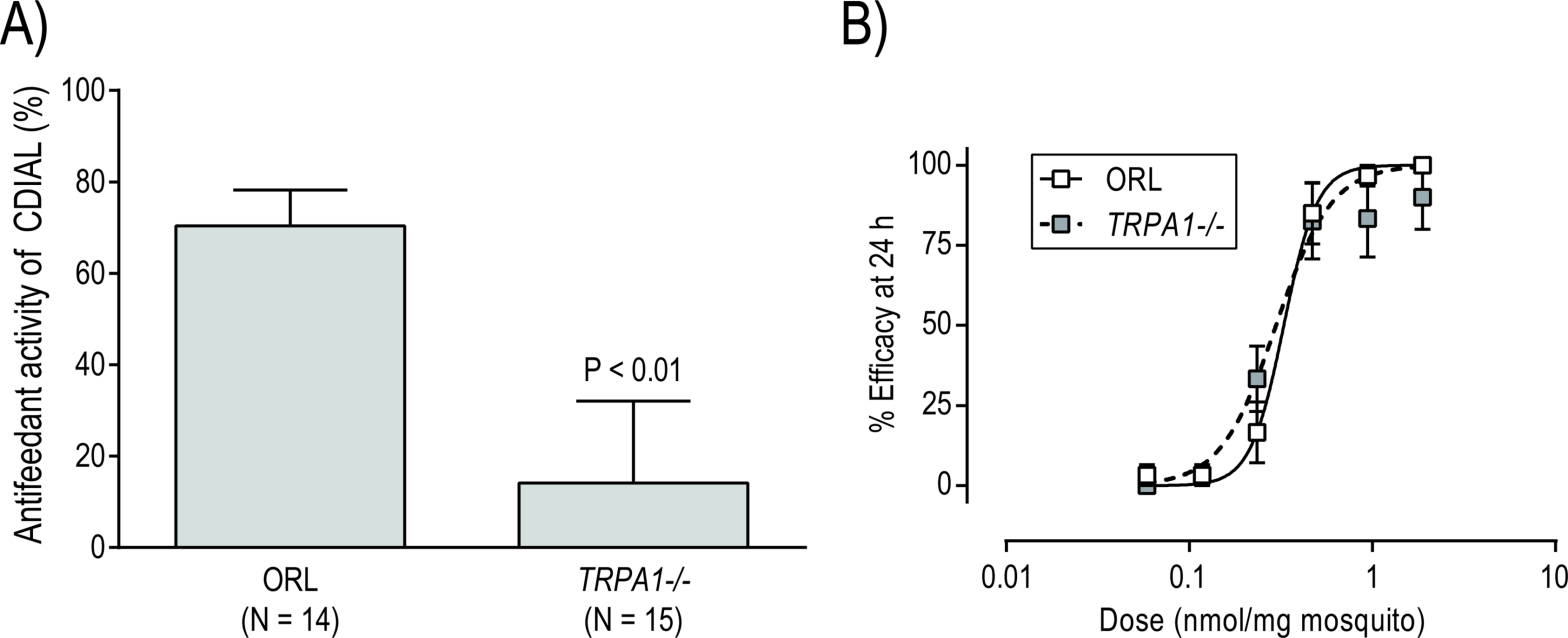
Effects of CDIAL on *TRPA1-/- Ae*. *aegypti*. A) Antifeedant activity of CDIAL (1.5 mM) in adult females of the parental ORL and *TRPA1-/-* strains of *A*. *aegypti*, as determined via the CAFE choice bioassay. Antifeedant activity was calculated as described in Fig 4. Values are means ± SEM; N = number of independent replicates of 5 mosquitoes each. P value indicates significant difference from ORL strain as determined via an unpaired t-test. B) Dose-toxicity relationship of CDIAL in adult females of ORL and *TRPA1-/- Ae*. *aegypti*. Efficacy was defined as the percentage of adults that were incapacitated (dead or flightless) within 24 h. Values are means ± SEM, based on 3–6 independent replicates of 10 mosquitoes per dose. The ED_50_ values and Hill slopes for the two strains were not significantly different from each other (P > 0.05; F-test). ORL: ED_50_ = 0.33 nmol/mg (95% C.I. = 0.28–0.38 nmol/mg); Hill slope = 4.77 (95% C.I. = 2.70–6.83). *TRPA1-/-*: ED_50_ = 0.30 nmol/mg (95% C.I. = 0.24–0.37 nmol/mg); Hill slope = 2.87 (95% C.I. = 1.16–4.58).

To confirm that CDIAL modulated TRPA1 in *Ae*. *aegypti*, we performed the CAFE choice assay on a mutant line of *Ae*. *aegypti* deficient in TRPA1 (*TRPA1-/-*) [31]. The parental Orlando (ORL) wild-type strain was used as a control. We hypothesized that the *TRPA1-/-* strain would exhibit a weakened antifeedant response to CDIAL compared to the ORL strain. Notably, the antifeedant activity of CDIAL was significantly weaker in the *TRPA1-/-* strain vs. the ORL strain (Fig 7A). Lastly, we tested whether adult females of the *TRPA1-/-* strain exhibited toxic resistance to CDIAL compared to the ORL strain. However, the potency (ED_50_) and steepness (Hill slope) of the dose-toxicity relationships of CDIAL were not significantly different between the two strains (Fig 7B).

## Discussion

The present study provides the first evidence that a bark extract of *C*. *fragrans* (CINEX), a Madagascan endemic plant used in traditional medicines, contains sesquiterpenes that are: 1) acutely toxic to larval and adult female *Ae*. *aegypti*, *An*. *gambiae*, and *Cx*. *pipiens* mosquitoes; and 2) antifeedant and repellent to adult female *Ae*. *aegypti*. Notably, CDIAL was primarily responsible for CINEX’s toxic, antifeedant, and repellent activities, while CFRAG, a dimeric form of CDIAL, also contributed to the antifeedant and toxic activity. CMOS did not possess detectable antifeedant or repellent activity, but elicited toxic effects against larval and adult female mosquitoes at high concentrations/doses.

Importantly, heterologous expression of *Ag*TRPA1 in *Xenopus* oocytes provided compelling evidence that the bioactivities of CDIAL and CFRAG were in part mediated via their activation of TRPA1. In both vertebrate and invertebrate animals, TRPA1 is a critical molecular sensor of temperature and noxious chemicals (e.g., electrophiles) [57–59]. CDIAL is structurally similar to polygodial (Fig 1), a drimane-sesquiterpene electrophile that is a known agonist of mammalian TRPA1 [37, 60]. CDIAL possesses two aldehyde functions (Ald1, Ald2); Ald1 is a more reactive electrophile than Ald2 because of a double bond between its alpha (C-8) and beta (C-7) carbons (S1 Fig). Relative to CDIAL, CFRAG is a weaker electrophile, because it only possesses Ald2 (Fig 1). As such, CFRAG was a weaker agonist of *Ag*TRPA1 compared to CDIAL. Likewise, CMOS is structurally similar to CDIAL with the exception of a γ-lactone replacing the two aldehydes (Fig 1). Although the lactone carbonyl is conjugated with the C-8:C-7 double bond, the lactone ring and absence of aldehydes make CMOS less electrophilic than CDIAL and CFRAG. As such, CMOS was an inferior agonist of *Ag*TRPA1 compared to CDIAL and CFRAG.

Electrophilic compounds activate TRPA1 channels by covalently binding to highly-conserved nucleophilic cysteine and lysine residues in the cytosolic NH_2_-terminal domain of the protein [61–63]; nearly all of the implicated cysteine and lysine residues are intact in mosquito TRPA1 [40]. Consistent with covalent binding of CDIAL and CFRAG to *Ag*TRPA1, the deactivation rates of *Ag*TRPA1 upon washing out either molecule was much slower than the corresponding activation rates. Rapid inactivation of *Ag*TRPA1 was only observed upon the addition of ruthenium red, which occludes the conductive pores of TRP channels [64]. In contrast to the asymmetric chemical activation/deactivation of *Ag*TRPA1 by CDIAL and CFRAG, the thermal activation/deactivation of *Ag*TRPA1 is symmetric and rapidly reversible [34]. These findings are consistent with the notion that TRPA1 channels possess independent mechanisms of thermal vs. chemical activation [32, 65].

The weaker antifeedant activity of CDIAL in the *TRPA1-/-* strain of *Ae*. *aegypti* compared to the parental ORL strain confirmed that CDIAL modulates TRPA1 in *Ae*. *aegypti*. A previous study similarly found that *N*-methylmaleimide, another electrophilic agonist of TRPA1 [40, 42, 61], elicited weaker antifeedant responses in the *TRPA1-/-* strain of *Ae*. *aegypti* vs. the parental ORL strain [31]. Assuming that the localization of TRPA1 in *Ae*. *aegypti* is similar to that in *D*. *melanogaster* and *Anopheles stephensi*, we suspect that CDIAL is detected by TRPA1-expressing neurons in the antennae and/or mouthparts [33, 34, 40, 66], leading to an antifeedant response. Consistent with this notion, CFRAG, a moderate agonist of *Ag*TRPA1, exhibited comparable antifeedant activity to CDIAL at the highest concentration tested (1.5 mM), but lost its antifeedant activity at 0.75 mM, a concentration at which CDIAL remained highly effective. Moreover, CMOS, a nominal agonist of *Ag*TRPA1, did not elicit detectable antifeedant activity at 1.5 mM. Thus, the relative antifeedant activities of the molecules in *Ae*. *aegypti* correlate with their respective ability to activate heterologously-expressed *Ag*TRPA1.

Of the sesquisterpenes tested, only CDIAL significantly repelled mosquitoes from feeding on a blood source, consistent with it being the most potent agonist of *Ag*TRPA1. A previous study has shown that exposure of adult female *An*. *stephensi* mosquitoes to allyl isothiocyanate, a volatile electrophile that activates TRPA1, dramatically inhibits their host-seeking behavior [66]. Thus, CDIAL may similarly disrupt the host seeking behavior of *Ae*. *aegypti*, leading to a lower propensity to blood feed. Further studies will be required to determine whether allyl isothiocyanate and CDIAL repel mosquitoes by activating TRPA1-expresing neurons in the antennae and/or proboscis [34, 66]. Remarkably, the repellency of CDIAL was comparable to DEET—the current gold standard for a repellent—despite an over 1.5-times lower molar amount of CDIAL vs. DEET used in the assays. Thus, our findings galvanize the notion suggested by others that TRPA1 agonists offer valuable potential molecules for the development of new repellents [31, 34, 40–43].

CDIAL was the most efficacious and/or potent sesquiterpene in eliciting toxicity to larval and adult female *Ae*. *aegypti*, but the mechanism by which it elicits toxicity appears to be independent of TRPA1. Notably, *TRPA1-/-* adult females did not exhibit toxic resistance to CDIAL compared to the parental ORL strain. Furthermore, CMOS, a nominal agonist of TRPA1, exhibited dose-dependent toxicity against larval and adult female mosquitoes, albeit only at high concentrations/doses. Thus, although TRPA1 is essential for the environmental sensing of CDIAL by mosquitoes, the activation of TRPA1 does not appear to be the primary mechanism of CDIAL’s toxicity. Instead, we propose that CDIAL, CFRAG, and CMOS disrupt cellular integrity, signaling, and metabolism according to their relative electrophilic activities; similar toxic consequences been attributed to other electrophilic sesquiterpenes [10, 37]. We can also ascertain that CDIAL is unlikely to modulate voltage-gated Na^+^-channels as part of its mechanism of action given the similar toxic potencies of CDIAL in pyrethroid-susceptible (LVP) and -resistant (PR) strains of *Ae*. *aegypti*; the PR strain contains knockdown resistance (*kdr*) mutations in voltage-gated Na^+^-channels that contribute to pyrethroid resistance [67]. The PR strain is also characterized by metabolic resistance, likely due to elevated mRNA levels of several cytochrome P450 monoxygenases and a glutathione *S*-transferase [67]. Thus, CDIAL may be a poor substrate of these detoxification mechanisms. These findings excitingly suggest that CDIAL could be developed into an insecticide for mitigating target-site and/or metabolic resistance in mosquitoes.

The results of the present study complement those from a recent study demonstrating that essential oils from the leaves and bark of the *C*. *fragrans* congener *C*. *madagascariensis* were toxic to larvae of *Culex quinquefasciatus* when added to their rearing water [30]. Although a specific toxic compound was not identified in the essential oils, the toxicity was attributed to its enrichment with monoterpenes (>75% composition) vs. sesquiterpenes (<17% composition), and CDIAL was not detected in the oil [30]. Thus, the genus *Cinnamosma* likely produces other natural products in addition to CDIAL with potential applications for vector control. If several common challenges associated with botanical pesticides can be overcome [1]—such as scaling up the available plant biomass via cultivation, efficiently isolating the active ingredients, and enhancing the shelf life of the active ingredients—and the active ingredients are shown to be non-toxic to vertebrates and beneficial insects, then *Cinnamosma* plants may represent a valuable botanical resource for developing next-generation vector control products to combat emerging mosquito-borne diseases.

## Supporting information

S1 Fig Examples of nucleophilic addition associated with the aldehyde functions of CDIAL. A) Addition of a nucleophile (Nu) at C-7 results in the movement of electrons (blue arrows) through Ald1 to Ald2. B) Addition of a Nu at Ald2 results in a less dramatic movement of electrons.
(PDF)

S2 Fig **Comparative toxicity of CDIAL in larval (A) and adult female (B) mosquitoes (*Cx*. *pipiens* and *An*. *gambiae*) 24 h after addition to the rearing water or application to the thoracic cuticle, respectively.** Values are means ± SEM based on 4–12 independent replicates per concentration/dose. The concentration/dose-toxicity relationships of CDIAL against *Ae*. *aegypti* from Fig 2 are superimposed (red-dotted lines) to facilitate comparisons. For larvae (A), efficacy was defined as the percentage that were dead within 24 h. For adult females (B), efficacy was defined as the percentage that were incapacitated (dead or flightless) within 24 h. For larvae, the EC_50_ values of CDIAL were 43.1 μM in *Cx*. *pipiens* (95% CI = 37.3–49.8 μM) and 96.7 μM in *An*. *gambiae* (95% CI = 67.3–139.0 μM); the Hill slopes were 2.09 in *Cx*. *pipiens* (95% CI = 1.57–2.605) and 0.74 in *An*. *gambiae* (95% CI = 0.47–1.00). For adult females, the ED_50_ values of CDIAL were 0.56 nmol/mg in *Cx*. *pipiens* (95% CI = 0.47–0.655 nmol/mg) and 0.16 nmol/mg in *An*. *gambiae* (95% CI = 0.08–0.30); the Hill slopes were 1.92 in *Cx*. *pipiens* (95% CI = 1.43–2.41) and 0.62 in *An*. *gambiae* (95% CI = 0.34–0.89).
(PDF)

S3 Fig Representative traces of membrane current (I_m_) in H_2_O-injected oocytes. Horizontal bars indicate the addition of 10 μM CFRAG or CMOS to the extracellular bath. Neither CFRAG nor CMOS noticeably changed I_m_.
(PDF)
