# Supplementary figures and images for: A natural agonist of mosquito TRPA1 from the medicinal plant Cinnamosma fragrans that is toxic, antifeedant, and repellent to the yellow fever mosquito Aedes aegypti
Source: PLoS Negl Trop Dis. 2018 Feb 9;12(2):e0006265. doi: 10.1371/journal.pntd.0006265 (PMC5823474; doi:10.1371/journal.pntd.0006265)

S1 Fig

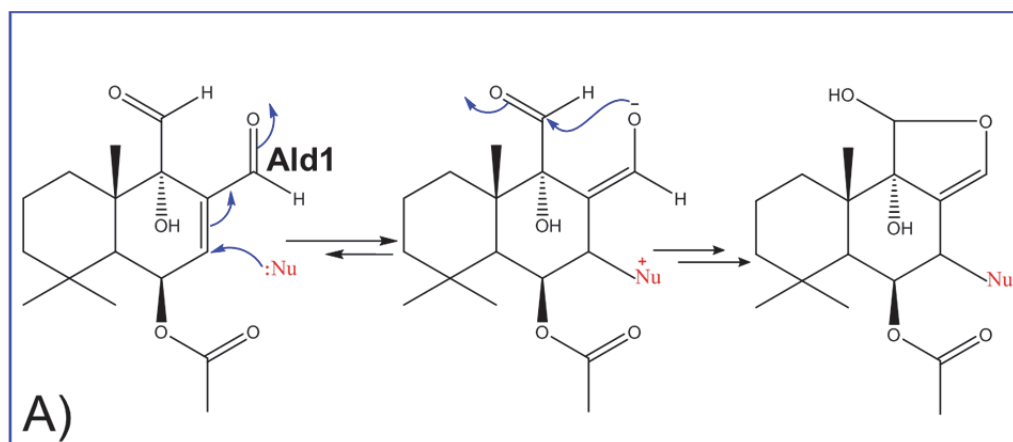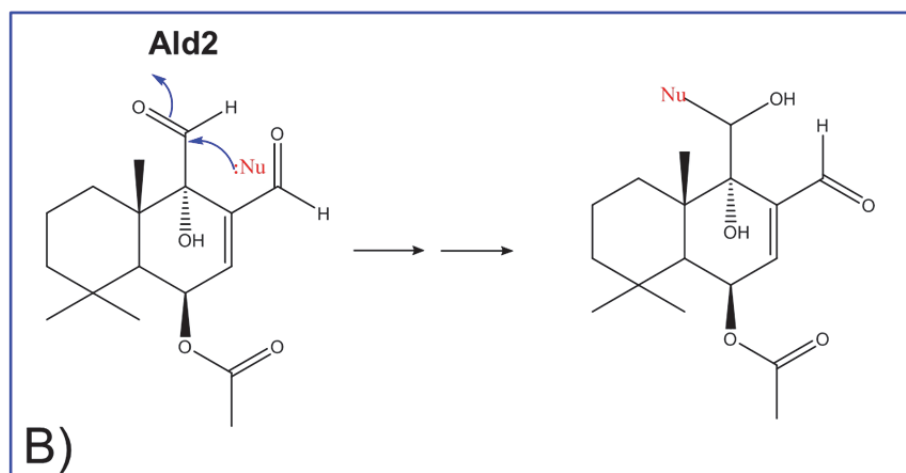

Supplement: S1 Fig — A) Addition of a nucleophile (Nu) at C-7 results in the movement of electrons (blue arrows) through Ald1 to Ald2. B) Addition of a Nu at Ald2 results in a less dramatic movement of electrons. (PDF) [file pntd.0006265.s001.pdf]

**S2 Fig**

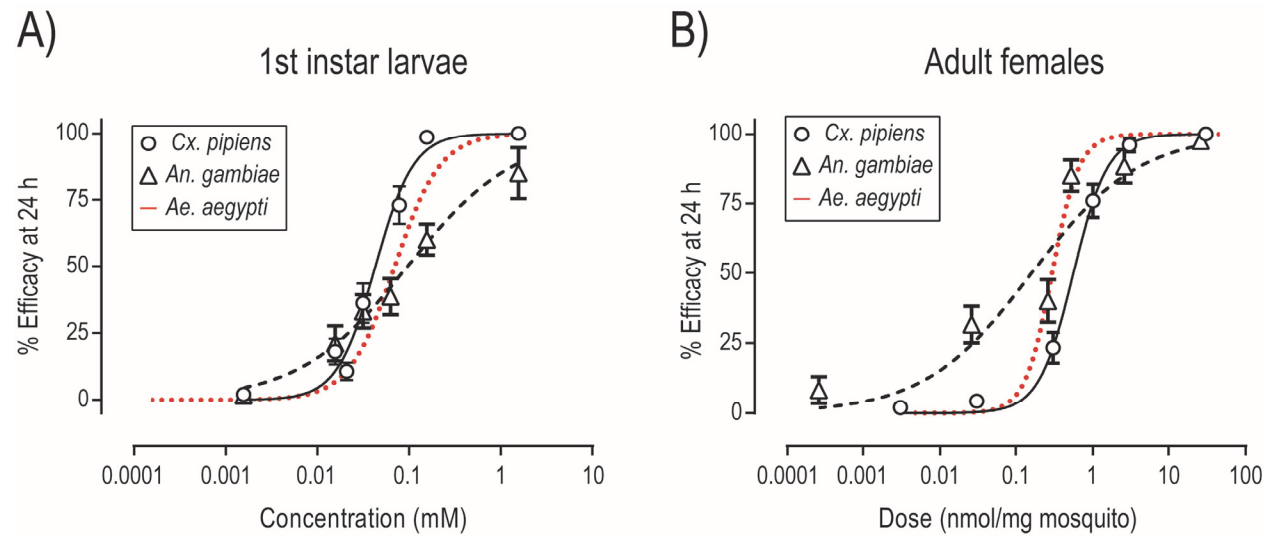

Supplement: S2 Fig — Comparative toxicity of CDIAL in larval (A) and adult female (B) mosquitoes (Cx. pipiens and An. gambiae) 24 h after addition to the rearing water or application to the thoracic cuticle, respectively. Values are means ± SEM based on 4–12 independent replicates per concentration/dose. The concentration/dose-toxicity relationships of CDIAL against Ae. aegypti from Fig 2 are superimposed (red-dotted lines) to facilitate comparisons. For larvae (A), efficacy was defined as the percentage that were dead within 24 h. For adult females (B), efficacy was defined as the percentage that were incapacitated (dead or flightless) within 24 h. For larvae, the EC50 values of CDIAL were 43.1 μM in Cx. pipiens (95% CI = 37.3–49.8 μM) and 96.7 μM in An. gambiae (95% CI = 67.3–139.0 μM); the Hill slopes were 2.09 in Cx. pipiens (95% CI = 1.57–2.605) and 0.74 in An. gambiae (95% CI = 0.47–1.00). For adult females, the ED50 values of CDIAL were 0.56 nmol/mg in Cx. pipiens (95% CI = 0.47–0.655 nmol/mg) and 0.16 nmol/mg in An. gambiae (95% CI = 0.08–0.30); the Hill slopes were 1.92 in Cx. pipiens (95% CI = 1.43–2.41) and 0.62 in An. gambiae (95% CI = 0.34–0.89). (PDF) [file pntd.0006265.s002.pdf]

S3 Fig

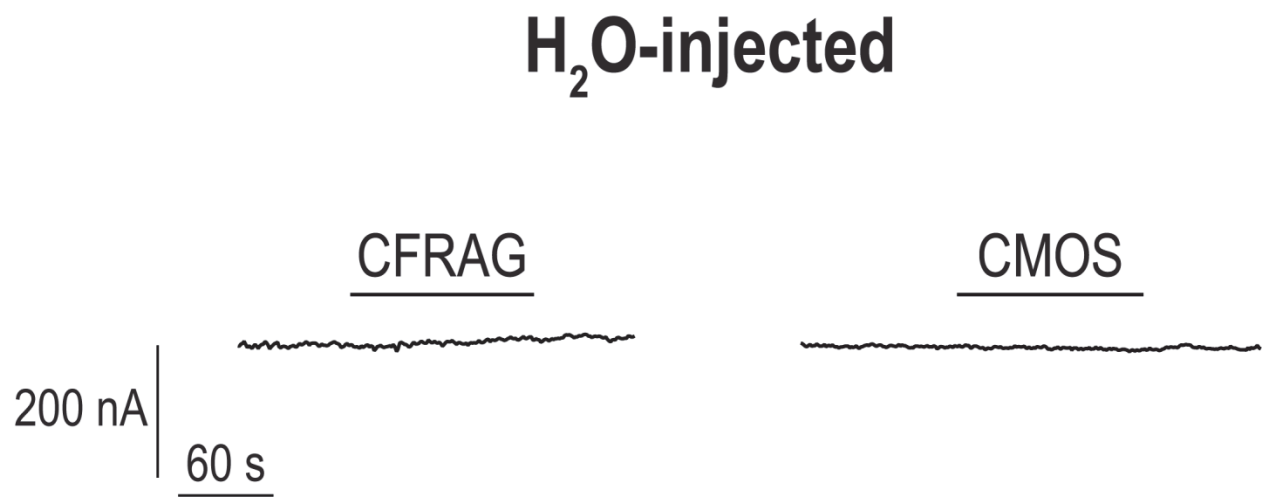

Supplement: S3 Fig — Horizontal bars indicate the addition of 10 μM CFRAG or CMOS to the extracellular bath. Neither CFRAG nor CMOS noticeably changed Im. (PDF) [file pntd.0006265.s003.pdf]
